# Frequency-dependent avoidance movement of glass catfish in response to sinusoidal electrical stimulation and associated spiking patterns of electroreceptors

**DOI:** 10.1242/bio.059796

**Published:** 2023-07-27

**Authors:** Yu Adachi, Katsumi Tateno

**Affiliations:** ^1^ Department of Life Science and Systems Engineering, Kyushu Institute of Technology, 2-4 Hibikino, Wakamatsu-ku, Kitakyushu 808-0196, Japan; ^2^ Department of Human Intelligence Systems, Kyushu Institute of Technology, 2-4 Hibikino, Wakamatsu-ku, Kitakyushu 808-0196, Japan; ^3^ Research Center for Neuromorphic AI Hardware, Kyushu Institute of Technology, 2-4 Hibikino, Wakamatsu-ku, Kitakyushu 808-0196, Japan

**Keywords:** Electroreception, Avoidance behavior, Local variability

## Abstract

The glass catfish is a freshwater fish with electroreceptors on its body surface. In this study, we investigated its behavioral response to sinusoidal electrical stimulation with a dipole wider than its body length and the spiking patterns of its electroreceptors. We observed that sinusoidal electric stimulation with a large dipole distance elicited in the glass catfish an avoidance movement whose frequency range is frequency-dependent. The movements were prominent in the frequency range between 10–20 Hz. When the stimulation strength increased, the movements were also found in the low-frequency range. In electrophysiological experiments, periodic interspike intervals of the electroreceptors were modulated by sinusoidal electrical stimuli. The stimulation introduced irregularity in the spiking patterns. The local variability of the spike modulations was significantly higher in the frequency range of 4–40 Hz and was particularly sensitive at 20 Hz. The avoidance movements and an increase in the local variability in the spike patterns were found around 20 Hz. Our results indicate that the glass catfish avoids sinusoidal electrical stimulation in a frequency-dependent manner, and this is associated with local modulations in the spiking patterns of the electroreceptors.

## Teaser

**Summary:** This paper shows that glass catfish exhibit frequency- and stimulus intensity-dependent avoidance behavior in response to alternating electric current stimulation.

## INTRODUCTION

Fish use electroreceptors to search for prey or detect predators and obstacles. For example, sharks search for small, buried fish using their electroreceptors (Kalmijn, 1971). A blindfolded catfish nibbled a metallic rod when 3 cm of it was immersed in water (Parker and van Heusen, 1917). Reportedly, paddlefish strike at electrode tips in the dark at low stimulus intensities (Wojtenek et al., 2001; Wilkens and Hofmann, 2007). Thus, electroreceptors help fish detect static electric fields (Paker and van Heusen, 1917; Gurgens et al., 2000; Peters and Bretschneider, 1972). In the case of the abovementioned catfish, an avoidance response was observed when 6 cm of a rod was immersed in water. Paddlefish also show avoidance movements in response to a metal rod exposed in water (Gurgens et al., 2000; Wilkens et al., 2002).

An environmental electric field often contains alternating electrical components. An alternating current is generated by organisms or obstacles under ordinary conditions. For instance, tadpoles or zooplanktons generate a relatively low-frequency periodic electric field due to muscle contractions (Peters and Bretschneider, 1972; Russell et al., 1999; Wojtenek et al., 2001). Fish possess a stationary electric field with fluctuations because respiration or other movements are superimposed. Catfish generate a direct-current (DC) component with an alternating electric field around their bodies (Peters and Bretschneider, 1972). Furthermore, encapsulated skate embryos reportedly freeze when electric fields are presented at 0.5 or 1 Hz (Sisneros et al., 1998). Thus, the ability to sense electric fields in fish can facilitate the search for prey as well as avoidance movements or predator detection.

*Kryptopterus bicirrhis* (glass catfish), a tropical nonelectrogenic transparent catfish, has ampullary electroreceptors (Wachtel and Szamier, 1969; Jørgensen, 1992). Ampullary receptor organs are distributed over their entire body. An ampullary receptor is comprised of a bottle-shaped receptor in the epidermis. The electroreceptor cells, which are located at the bottom of the receptor, are innervated by lateral line nerves (Jørgensen, 1992; Roth, 2006). The afferent nerves have a spontaneous spike rate of approximately 60 Hz without an external stimulus input. Glass catfish can passively perceive environmental electric fields, and sinusoidal electrical stimulation induces frequency modulation in the spike trains of nerve terminals. Electroreceptors are sensitive to low-frequency (<20 Hz) electric stimulation (Bretschneider et al., 1985). Alternatively, glass catfish show avoidance behavior in response to static or to 50 Hz of alternating magnetic stimulation (Krishnan et al., 2018; Hunt et al., 2021). Yet, their behavioral responses under an electric field remain unknown.

Knowing the responses of electroreceptors to electrical stimuli and the associated behavioral responses of fish promotes understanding of the mechanism of information processing related to electroreception, which addresses the question of what changes in neural activity patterns contribute to behavioral changes. The spiking patterns of afferent nerves might encode the detailed features of environmental stimulation. Doiron et al. (2003) described how environmental electrical stimulation from different sources generated various temporal patterns in the nerve spikes of an electrogenic knifefish. Stimulation through environmental electric signals changes the spiking patterns of the electroreceptors, which subsequently induce associated movements. The temporal patterns of electroreceptor responses in glass catfish are poorly understood, although electrical stimulation might affect their swimming behavior.

Sinusoidal electrical stimulation to glass catfish shortens the interspike interval (ISI) in one half period and prolongs the ISI in the other half (Bretschneider et al., 1985). Bursting patterns are considered to contribute to reliable signal transmission (Izhikevich et al., 2003) and are often found in electroreceptors and their associated cells (Wilkens et al., 1997; Neiman and Russell, 2002; Oswald et al., 2004; Lankheet et al., 2012). *Daphnia* or Ornstein-Uhlenbeck Gaussian noise induce bursting in the electroreceptors of paddle fish (Wilkens et al., 1997; Neiman and Russell, 2002). In the electrosensory lateral line lobe, pyramidal cells respond to stimuli in bursts. Those intervals in the bursts correlate with the intensity of its stimulus upstroke (Oswald et al., 2007). ISI modulations of catfish in response to electrical stimulation have been reported for their frequency dependence, but not for their dependence on stimulus intensity.

In this study, we investigated the swimming behavior of glass catfish in response to sinusoidal electrical stimulation and elucidated the spiking patterns of electroreceptors and their afferent nerves associated with this stimulus. Additionally, we discussed swimming behavior in response to electrical stimuli on the basis of simulations of electrostatic field gradients.

## RESULTS

### Swimming behaviors in response to sinusoidal electrical stimulation

Fig. 4 shows the swimming locus of a fish in the annular aquarium under recording conditions with or without electrical stimulation. In the no-electrical-stimulation condition, the fish turned around, and their swimming locus was basically circular (left in Fig. 4A). Under the electrical-stimulation condition, the fish swam back and forth in the aquarium without entering the lower section (right in Fig. 4A). The stimulation frequency was 20 Hz, and the current's peak-to-peak amplitude was 500 nApp. Fig. 4B shows the fish location as an angle of the annular aquarium. The angle was added (2π) after one round and was not reset. The stimulation electrode's location is highlighted with dashed lines. Seven fish are shown in different colors. In the left and right figures in Fig. 4B, the same colors represent data from the same fish. Without stimulation, the glass catfish circled in the aquarium. When stimulated, some (indicated by black or green lines) moved back and forth between the dashed lines. The absolute value of the angle difference between the angle θ_60_ at the end time of stimulation minus the angle θ_0_ at the start time of stimulation was statistically verified (Fig. 4C). There were no significant differences between the conditions [paired *t*-test, *n*=7, *t*(6)=1.276, *P*=0.249]. However, when stimulated, the angle difference tended to be smaller. Fig. 4D shows the distance traveled for each sampling time (1 s). The same fish as in Fig. 4B are presented by the same colored dots. The distance traveled in the vicinity of the electrode was longer than that around π/2, suggesting that the fish hurried past the stimulation electrode. The traveled distances per second at the upper and lower half of the experimental tank were quantified (Fig. 4E). It was determined that the fish moved significantly faster in the lower half of the experimental tank [Welch's *t*-test, *t*(96.919)=6.090, *P*<0.001].

Fig. 5A–F are all-point histograms of the angles collected from the seven fish, compared for six stimulus conditions. Without electrical stimulation, the fish were equally present in all the positions in the aquarium (Fig. 5A and D). When 4 Hz of the 250-nApp stimulation was applied, the bin counts slightly decreased near the electrodes, but this was not apparent (Fig. 5B). For 20 Hz, the bin counts decreased near the stimulating electrode and increased away from it (Fig. 5C). When 500-nApp stimulation was applied, the bin counts increased in the upper section (0−*π* rad and 2π−3π rad) at both 4 Hz (Fig. 5E) and 20 Hz (Fig. 5F). Fig. 5G shows the cumulative distribution of the distance from the outer electrode. Since the distribution was approximately uniform in the control experiments, the cumulative frequency increased linearly (skewness=−0.14, dashed line in Fig. 5G). When the electric stimulation was given, the distribution skewed to the left. The skewness of the frequency distribution was greater at 20 Hz (skewness=−1.3) than at 4 Hz (skewness=−0.64). These results indicate that the sinusoidal electric stimulation caused the glass catfish to swim away from the electrodes during the sinusoidal stimulation.

The angular distribution of the positions of the glass catfish was evaluated by circular statistics (Table 1). The median angles and the standard deviations were also included. First, we examined the angular data drawn from a circular uniform distribution by means of a Kuiper one-sample test (*P*<0.01). A null hypothesis means that the angular distribution is a circular uniform distribution. A significant difference means that the angular distribution is biased. At 250-nApp stimulation, the angular distribution was not considered to be a circular uniform distribution at 10 Hz, 20 Hz, and 50 Hz. At 400-nApp stimulation, significant differences were found in the frequency range between 0.5 Hz and 40 Hz. At 500-nApp stimulation, significant differences were found in the frequency range between 0.5 Hz to 100 Hz. As the stimulus intensity was increased, the frequency band where the distribution showed a non-uniform distribution was widened. Second, a Watson's goodness-of-fit test was applied for testing the null hypothesis that the angular data was drawn from a von Mises distribution, which is a unimodal distribution. In this case, significant differences mean that angular distributions were not in a von Mises distribution. Several distributions were neither a circular uniform distribution nor a unimodal one. At 250 nApp, the median angles were 1.4 at 10 Hz and 1.6 rad at 20 Hz. This shows that the fish frequently stayed on the side opposite the stimulating electrode when the stimulation frequency was 10–20 Hz. Stimulation of 50 Hz caused a von Mises distribution, but the medial angle was 2.96 rad with a relatively large SD (= 1.92). At 400 nApp, the angular distributions of 4 or 10 Hz were significantly different from the unimodal distributions. However, the medial angles were 1.56 and 1.34 rad, respectively.

**Table 1.**
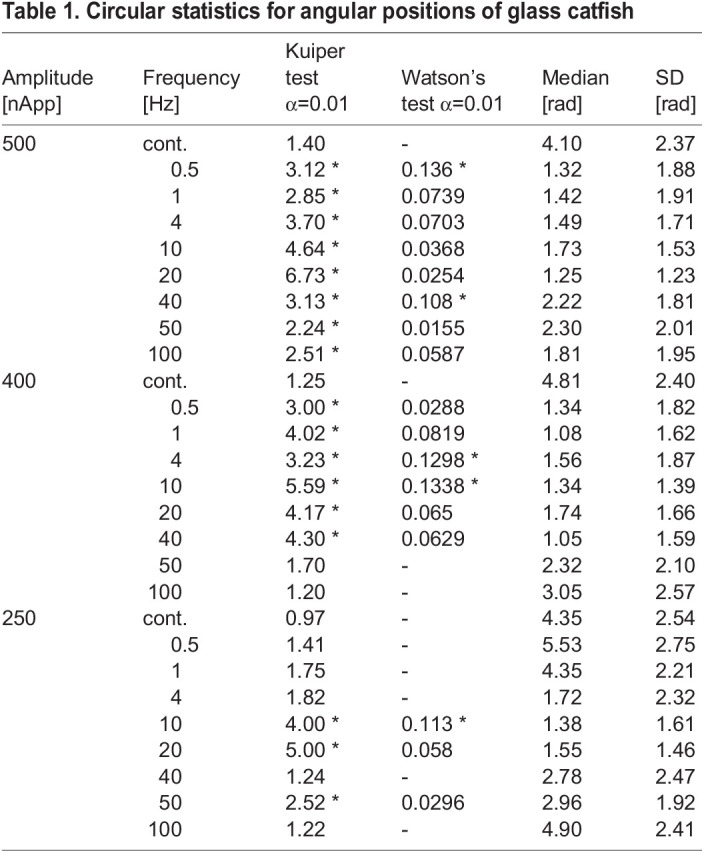
Circular statistics for angular positions of glass catfish

Fig. 6 shows histograms of the mean cumulative dwelling times in the lower section. The results were analyzed using a one-way analysis of variance (ANOVA) with Dunnett's test for comparisons with the control group. At 250-nApp stimulation, significant avoidance behavior was observed at 10 and 20 Hz (Fig. 6A) (one-way ANOVA with Dunnett's test, *n*=7, *F*(8, 48)=5.12, *P*<0.001). At 400-nApp stimulation, the mean cumulative dwelling time was significantly below the control group at 1 Hz and 10–40 Hz (Fig. 6B) (one-way ANOVA with Dunnett's test, *n*=7, *F*(8, 48)=3.746, *P*=0.002). At 500-nApp stimulation, the mean cumulative dwelling time was particularly short at 0.5–40 Hz and 100 Hz (Fig. 6C) [one-way ANOVA with Dunnett's test, *n*=7, *F*(8, 48)=8.168, *P*<0.001]. These results indicated that the glass catfish avoided the lower section under low-frequency stimulation. Those avoidance movements were frequency- and amplitude-dependent.

Simulations of the electrostatic field in the annular aquarium are shown in Fig. 7. The electrodes were located on the inner and outer walls. As the electrical stimulation was a low-frequency alternating current, the electric field potential was calculated as DC. Fig. 7 shows the case where the cathode was on the inner wall (left column) and the case where the cathode was on the outer wall (right column). The upper row is for high-voltage conditions (1.5 mV), and the lower row is for low-voltage conditions (0.75 mV). Under both conditions, the field potential spread concentrically from the electrodes. The field potential decreased with the distance from the electrodes. The potential gradient near the electrodes was steep and became gentle with distance. When the cathode potential was high, the equipotential lines were densely spaced even at a distance. In the upper section, the field potential fluctuated as the position of the cathode was swapped, but there was no potential gradient.

### Electroreceptor responses to sinusoidal electrical stimulation

The afferent nerves fired spontaneously without external stimulation, and the instantaneous firing frequency was the inverse of the ISIs. Electrophysiological data were collected from 21 of the electroreceptor organs of six fish. The spontaneous frequency of the nerve spikes (21 of the electroreceptor organs) was 52.2–113.9 Hz. Fig. 8A shows that the response of the afferent nerve spikes was modulated by the sinusoidal electrical stimulation (20 Hz, 500 nApp). ISIs were shortened in half of the sinusoidal periods and prolonged in the other half. Thus, the sinusoidal stimulation caused the ISIs to wax and wane. The hyperpolarized current interrupted the spiking, causing a burst in the spikes. Fig. 8B shows the local variation *Lv* as a function of the stimulation frequency. The *Lv* was frequency-dependent. As the stimulus amplitude increased, the frequency range of the large *Lv* also increased. With a 400-nApp stimulus amplitude, *Lv* was significantly higher in the frequency range between 10 Hz and 40 Hz, compared with the control [one-way ANOVA, *n*=21, *F*(7,140)=22.060, *P<*0.001]. With a 666-nApp stimulus amplitude, *Lv* became larger in the range of 4 to 40 Hz (one-way ANOVA, *n*=21, *F*(7,140)=41.148, *P*<0.001). In a frequency range of 80 Hz or higher, *Lv* did not increase. Fig. 8C shows the mean spike rate as a function of the simulation frequency. Alternatively, the mean spike rate was not dependent on the stimulation frequency. No significant differences were found at any of the stimulation frequencies [one-way ANOVA, *n*=21, *F*(7,140)=1.529, *P*=0.162 at 400 nApp; *F*(7,140)=1.482, *P*=0.178 at 500 nApp; *F*(7,140)=3.585, *P*=0.001 at 666 nApp]. Our results indicate that the spiking patterns encoded the input amplitude information, especially in a low stimulation frequency range, although the spike rate did not.

Fig. 9 shows ISI histograms. The ISIs for spontaneous spiking had a unimodal histogram (Fig. 9A). At 20 Hz, the ISI histogram looks bimodal (Fig. 9B). The Silverman test, a nonparametric multimodal test, was used to test the mode number of the histograms. Fig. 9C shows a summary of the modes indicated by the test. Bimodal or trimodal histograms were observed between 10 Hz and 40 Hz for 400 nApp and between 4 Hz and 20 Hz for 500 nApp. Some data were considered trimodal. In the trimodal histograms, the number of spikes and ISIs fluctuated in each periodic cycle, so the pause length between bursts fluctuated as shown in Fig. 8A. These histograms mean that the spike patterns were bursting. For 20 Hz at 666 nApp, a second bump appeared, but the bimodal distribution was less clear due to fluctuations in the spikes (data not shown). Therefore, those histograms were determined to be unimodal in the Silverman test. The same results were obtained at 40 Hz for 500 nApp and 666 nApp.

## DISCUSSION

### Spike response patterns and avoidance movements

The sinusoidal electrical stimulation modulated the ISIs of the afferent nerve spikes of the electroreceptors in the frequency range below the spontaneous spike rate. The ISI patterns changed with the amplitude or the input frequency of the stimulation. The passband of the afferent nerves corresponded to that found in a previous study (Bretschneider et al., 1985). In our study, the glass catfish exhibited avoidance or escape movements in response to the sinusoidal electrical stimulation in the sensitive input frequency range. Thus, ISI patterns might be associated with the avoidance movements of fish.

In our behavioral experiments, for 400-nApp stimuli, the glass catfish maintained a certain distance from the stimulation electrodes in the frequency range of 1–40 Hz. Glass catfish avoided magnetic stimulation at 50 Hz in a paper by Krishnan et al. (2018). As there is a difference between electric and magnetic stimulation, it is difficult to compare those stimulations directly, but the difference in frequency is probably a difference in stimulus amplitude.

The afferent nerves of glass catfish possess filtering properties (Bretschneider et al., 1985), and the effects of the alternating-current stimulation were limited in the frequency range. In these electrophysiological experiments, ISI modulation to sinusoidal electric stimulation was effective in a frequency range of approximately 4–20 Hz. In their paper, the stimulation amplitude dependence of the passband on the sinusoidal electric stimulation was not reported. In our electrophysiological experiments, for 400-nApp stimuli, *Lv* peaked at 20 Hz, with a significantly higher *Lv* in a wider range (10–40 Hz). Their high *Lv* range extended to 4–40 Hz with an increasing stimulus amplitude (666-nApp). The frequency range of the avoidance movements widened with an increase in the stimulation amplitude. Both the avoidance behaviors and the electroreceptors were sensitive around 10–20 Hz, and their frequency range widened as the stimulus amplitude increased. These results suggest that avoidance behaviors were elicited by electrical sinusoidal stimulation through electroreceptors.

Even though the mean spike rate was invariant in all the frequency ranges, the *Lv* values were frequency-dependent, and they increased with the rise in the stimulus amplitude. As the stimulus amplitude increased, the frequency band showing high *Lv* values expanded into the low-frequency range. Larger local variations were found around 10–20 Hz. Those results suggest that the local variability of the peripheral nerves correlates with avoidance movements in glass catfish.

The results of the electric field simulations suggest that glass catfish may avoid regions with steep potential gradients. The dwelling time in the upper section of the aquarium in response to the electrical stimulation was longer, but there were oscillations in the electric potential as well. However, the potential gradient was quite small in the upper section. Because glass catfish have electroreceptors on their entire body, a steep gradient in the field potential means that the head and tail receive different potential inputs. It is suggested that those different potential inputs result in different spike fluctuations for different receptors.

### Signal pathway from electroreceptors to brain

In glass catfish, since many electroreceptor organs are distributed all over their bodies, local and global electrical stimulations may produce different spike patterns. The afferent nerves transmit signals to the brain in glass catfish, and the afferent nerves of the anal fin fuse with the ventral ramus of the lateral line nerves (Roth, 1993). No feedback pathway to the electroreceptors and the projections of the lateral line nerves in the brain has been previously reported. If there is no sensitivity adjustment by feedback, ISIs modulated by gradients of the electric field are directly transmitted to the central nervous system. Stimulation with a large electrode spacing affects the entire body, thus stimulating many electroreceptors. At 10–20 Hz, as an ISI is modulated even at small amplitudes, glass catfish are capable of capturing voltage gradients.

### What are glass catfish avoiding?

Low-frequency sinusoidal electrical stimulation induced escape reactions or movements to avoid the electrodes. Our results show that low-frequency sinusoidal electrical stimulation is an undesirable signal for glass catfish. In this study, the dipole distance was longer than the glass catfish's body length. Fish generate an electric field where fluctuations are superimposed (Peters and Bretschneider, 1972). Kalmijn (1972) divided the electric field emitted by teleosts into a DC, AC field below 20 Hz and an AC field above 20 Hz. The electroreceptor response of glass catfish is less sensitive to DC but more sensitive to responses around 20 Hz. It is well within the frequency range that potential predators can emit. Electric fields greater than the dipole size might belong to a large fish, which could be a predator. Thus, we believe that the avoidance movements of glass catfish in response to electrical stimulation are associated with the detection of large predators. Since there are other fish with similar electrosensory frequency characteristics (Asano and Hanyu, 1986; Peters et al., 2001), a large dipole distance potentially induces avoidance behavior.

Fish behaviors depend on stimulus properties, such as frequency, amplitude, and dipole size (electrode spacing). Electroreceptors might facilitate the search for prey. In this study, the glass catfish did not nibble or attack the electrodes. The mean dwelling times in the lower section were not significantly longer than the control groups. The fact that the glass catfish did not stay near the electrodes may have something to do with the wide distance between the electrodes. Since the electrode distance was wider than the fish's body length, the glass catfish did not recognize the electric field as prey. Peters et al. (2001), while declining to say that the data were unpublished, reported that small weak dipole sources with 1 Hz are attractive, whereas large strong dipoles with 10 Hz are repulsive. Different sizes of dipoles potentially induce different behavior in glass catfish. *Daphnia* of 1–2 mm in length emit electrical signals of 1–15 Hz (Freund et al., 2002). Even in *Daphnia magna*, the length is 3–5 mm. As their electrical signal is weak, it cannot be detected without getting close. Such a weak small dipole distance stimulates the electroreceptors in the epithelium only locally. If the stimulus properties are equivalent to those of zooplankton, a behavior different from that of avoidance can be expected. Further research is needed on the possible dependence on electrode spacing and pattern as well as stimulus frequency and amplitude.

Glass catfish potentially generate alternating electric fields all around their bodies through the muscle contractions required for swimming. The sarcomere length changes during swimming at approximately 4 Hz (Lieber et al., 1992), which is in the frequency range of avoidance. However, the sensitivity to relatively 4 Hz is low. The glass catfish swarmed under a bright light, which might indicate that the strength of the electric field generated by the glass catfish is weak or that visual inputs can suppress the escape reaction.

We found that glass catfish avoid alternating electric stimuli in a frequency-dependent manner. The dipole distance was wide enough so that only avoidance behavior could be observed, although if the dipole distance were narrowed to mimic *Daphnia*, predatory behavior might be encouraged. If so, glass catfish can determine their behavior depending on the dipole's frequency, size, and intensity.

### Species difference in behavioral responses to frequency responses of electrosensory primary afferents

Skate embryos (Sisneros et al., 1998) and shark embryos (Kempster et al., 2013) cease their respiratory gill movements when detecting predator-mimicking electric fields. Their frequency range is below 1 Hz. In skate embryos, the peak gain of the electrosensory primary afferents is 1–2 Hz (Sisneros et al., 1998). The avoidance behavior of glass catfish shows a higher and wider frequency range than the freezing response of skates or shark embryos. The electrosensory primary afferents of glass catfish show high *Lv* values in the frequency range between 4–40 Hz. Differences in the frequency range of primary afferents may contribute to differences in the frequency response of avoidance behavior. The ISI distributions of electrosensory primary afferents in skate embryos differ among ontogenetic stages. Although there are no reports to date on the predator avoidance of adult skates to electrical stimuli, it may be that with growth, the spontaneous firing frequency of the primary afferents increases and behavioral responses change. Skates and sharks grow to large sizes and may no longer need to exhibit aversive behavior. Glass catfish, on the other hand, grow only to about 10 cm in length and may exhibit avoidance behavior in response to electrical stimuli.

## MATERIALS AND METHODS

The experimental procedures used in the electrophysiological and behavioral experiments are described below. All the experimental protocols were approved by the Animal Institutional Review Board of the Kyushu Institute of Technology.

### Behavioral responses to sinusoidal electrical stimulation

#### Recording procedures for behavioral responses to sinusoidal electrical stimulation

**Fig. 1.**
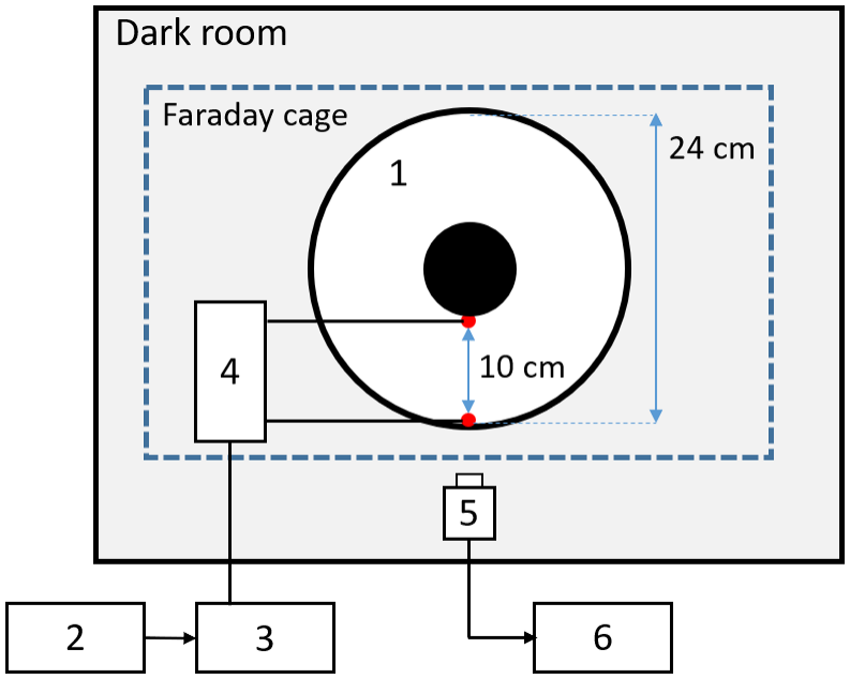
**Experimental installation for swimming behavior experiments.** Stimulation electrodes were set in lower half of annular aquarium (1). Sinusoidal electrical stimulation was generated by sine-wave generator software (2) and filtered by bandpass filter (3). Filtered voltage signals were converted into current by V-I converter (4). Swimming behaviors were captured by high-speed camera (5) installed under aquarium and controlled by computer (6) in which movies were stored.

Fig. 1 shows the experimental installation used for observing the behavior of glass catfish. The glass catfish (*n*=7) were 6.8–7.9-cm long. They swam freely in an annular aquarium (inner diameter=24 cm), and their behavioral responses to weak electrical stimulation were observed. A plastic tube (outer diameter=4 cm) was glued at the aquarium's center. The water route was 10-cm wide. The aquarium's external part was covered with a heat-insulating sheet. The water in the aquarium was maintained at a depth of 5 cm (2.4 L) to approximate its internal part as a two-dimensional plane. The water conductivity was 27.5–47.1 mS/m, and the temperature was 25°C–26.5°C. The experiments were conducted in a dark room. The aquarium was illuminated with infrared light-emitting diodes (LEDs) (940 nm, OptoSupply). Lighting is important for accurate detection by our tracking software. Fifty-six infrared LEDs, which were arranged in 7×8 rows, illuminated the entire aquarium as evenly as possible. Since LEDs tend to be spotlights and interfere with the detection of fish, we diffused their light so that it shined evenly throughout the aquarium. A diffuser film (Scotchcal Film, 3 M) was placed under the infrared LEDs to ensure even light. Another diffuser film was placed on the aquarium's lid. We recorded the swimming behavior of the glass catfish using a high-speed camera (Kissei Comtec Co., Ltd).

#### Electrical stimulation

An electrical stimulus was applied by a pair of electrodes (silver chloride electrode; KU204-074, Unique Medical Co., Ltd), fixed in a section in the lower half of the aquarium. The tip of each electrode was set 2.5 cm above the aquarium's bottom. The distance between the electrodes was 10 cm. A sinusoidal voltage wave was generated by a computer, and the DC and high-frequency components caused by digital-analog conversion were eliminated with a bandpass filter (3625 dual-channel programmable filter, NF Electronic Instruments). The cutoff frequencies of the high- and low-pass filters were 0.01 and 1000 Hz. The stimulation voltage was converted into current through a V-I converter circuit (1000 nA/V). The peak-to-peak amplitudes used for the stimulus current were 250, 400, and 500 nApp. The stimulus frequencies were 0.5, 1, 4, 10, 40, 50, and 100 Hz in a random order. Additionally, fish behaviors without any stimulus were recorded as a control. The recording was made for 60 s under each stimulus condition. The water in the experimental aquarium was drained and refilled before every experiment.

#### Analysis of swimming behavior

**Fig. 2.**
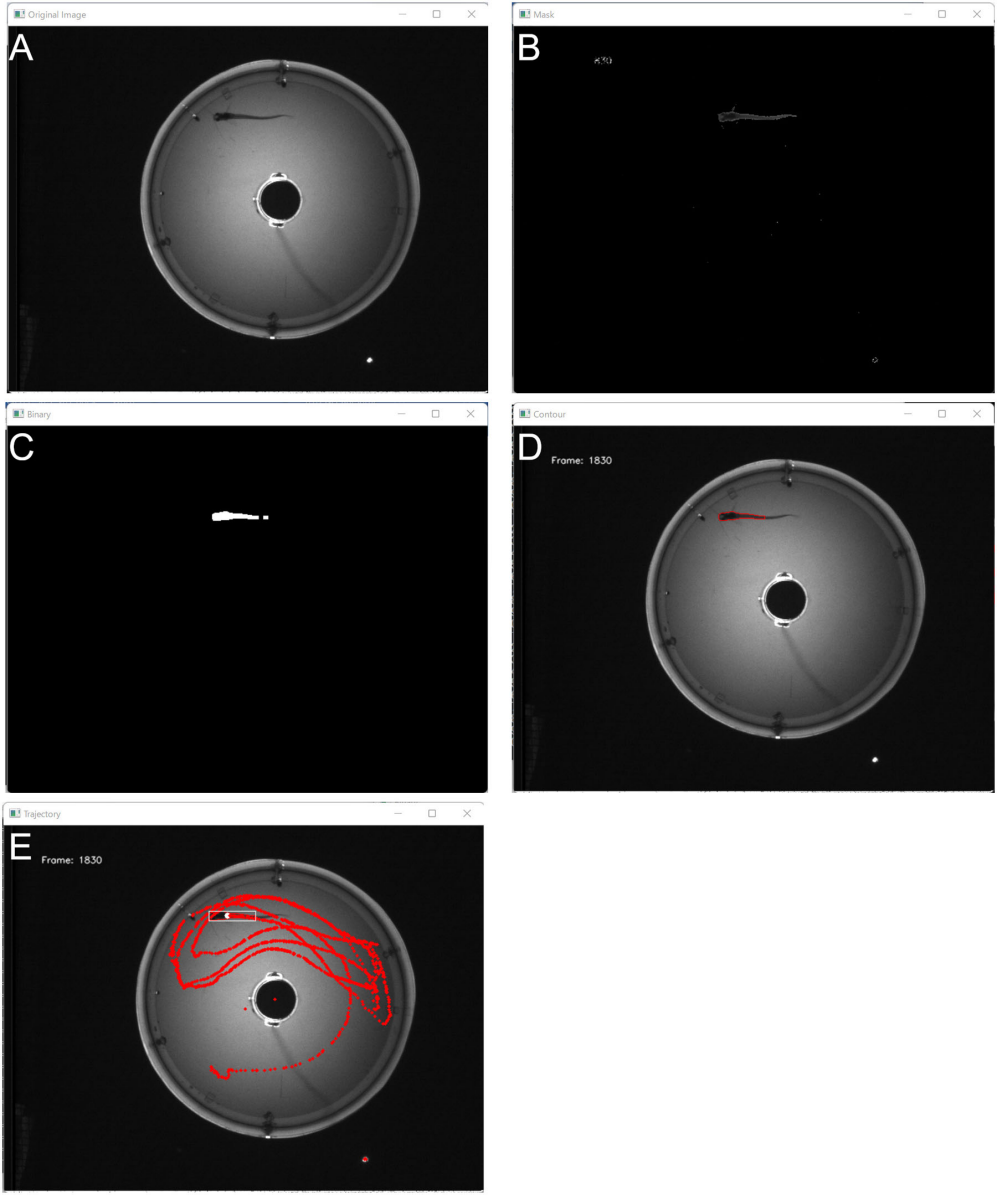
**Image processing method for tracking fish.** (A) Original image. (B) Background subtraction by backGroundSubtractor function of OpenCV. (C) Binary image. Masked images were eroded and dilated twice and binarized. Since the glass catfish's tail is translucent, their head is mainly accentuated. (D) Contour image, which surrounds largest chunk of binarized image with contour lines. (E) Trajectory. Largest chunk's mass-center is plotted (red dots). Incorrectly detected points were corrected visually. Here, position was estimated every 1/30 s.

After obtaining the recordings, the fish locations were determined using custom-made tracking software (Fig. 2). Our tracking software works in a semi-automatic way. After applying the software, the position information for analysis was extracted every 30 frames. All plotted positions were visually verified, and incorrect plots were corrected manually. This saves labor compared with manual work alone and achieves the same accuracy as manual work.

Fig. 2A is an original image. Using the tracking software, the fish locations were determined through background subtraction (Fig. 2B). The result obtained after image subtraction was assumed to be the image of a fish head because the body is transparent. Dilation and erosion of the binary images were repeated twice to suppress background noise. Gray images were converted into binary images (Fig. 2C). Contours were identified in each binary image, and contour areas of a certain size were extracted (Fig. 2D). The mass center of the extracted contour area was considered the fish's location (Fig. 2E). After applying the tracking software, any incorrectly plotted locations were visually corrected. The camera's frame rate was 30 fps. We analyzed the location of the fish once every 30 frames. In short, the sampling rate was 1 Hz. Each fish location was plotted to polar coordinates, the origins of which were the center of the aquarium.

If the fish avoid electric stimulation, the angular distribution of the fish positions should not be a circular uniform distribution. The fish may also stay away from the stimulation electrodes. Circular statistics analysis was applied to the extracted angular data using R. A Kuiper's test to assess the bias of the angular distribution. For data that were not in a circular uniform distribution, a Watson's goodness-of-fit test was performed for the von Mises distribution. In addition, to determine whether the fish stayed distal or proximal to the electrodes, the aquarium was divided into two equal sections on a two-dimensional plane: upper and lower. The section where the electrodes were installed was designated as the lower half section in which the dwelling time was analyzed. We prepared histograms of the dwelling times in the lower section and used a one-way ANOVA followed by Dunnett's two-tailed test to determine whether the time spent by the glass catfish in the lower section was below the control. A *P*-value <0.05 was considered significant.

#### Estimation of electrostatic field generated by electric stimulation

The finite element method was used to simulate the electric field in the aquarium. Although the electrical stimulation in this study was AC, the stimulation frequency was relatively low, so the electric field potential *φ*(***x***) was calculated under electrostatic conditions. The electric field was calculated as Poisson's equation:

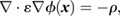
where ε is the electrical permittivity, and ρ is the charge density. We estimated the electrostatic field in the annular aquarium using free software, Finite Element Method Magnetics (FEMM, https://www.femm.info/wiki/HomePage), produced by David Meeker. FEMM was used to simulate the electrostatic field at constant voltage. The problem was a planar problem and simulated the electric field at an aquarium scale similar to the behavioral experiment. Electrodes with a radius of 5 mm were placed on the inner and outer walls. For applying the finite element method, we measured the electric potential between the electrodes in the actual aquarium. Since a V-I converter was used in our experiments, the voltage applied to the electrodes depended on the electric conductivity of the water. When the electric conductivity was 40 mS/m and the stimulus intensity was 500 nApp, the voltage applied to the electrodes was approximately 3 mVpp, so in the electrostatic field calculation, the voltage was set to 3 mVpp. The inside of the aquarium was set to a relative electrical permittivity of 80.4, equivalent to water at 20°C. Fixed voltage boundary conditions (*V*=0 mV) were applied.

### Electrophysiological responses of electroreceptors to sinusoidal electrical stimulation

#### Nerve spike recording procedures

**Fig. 3.**
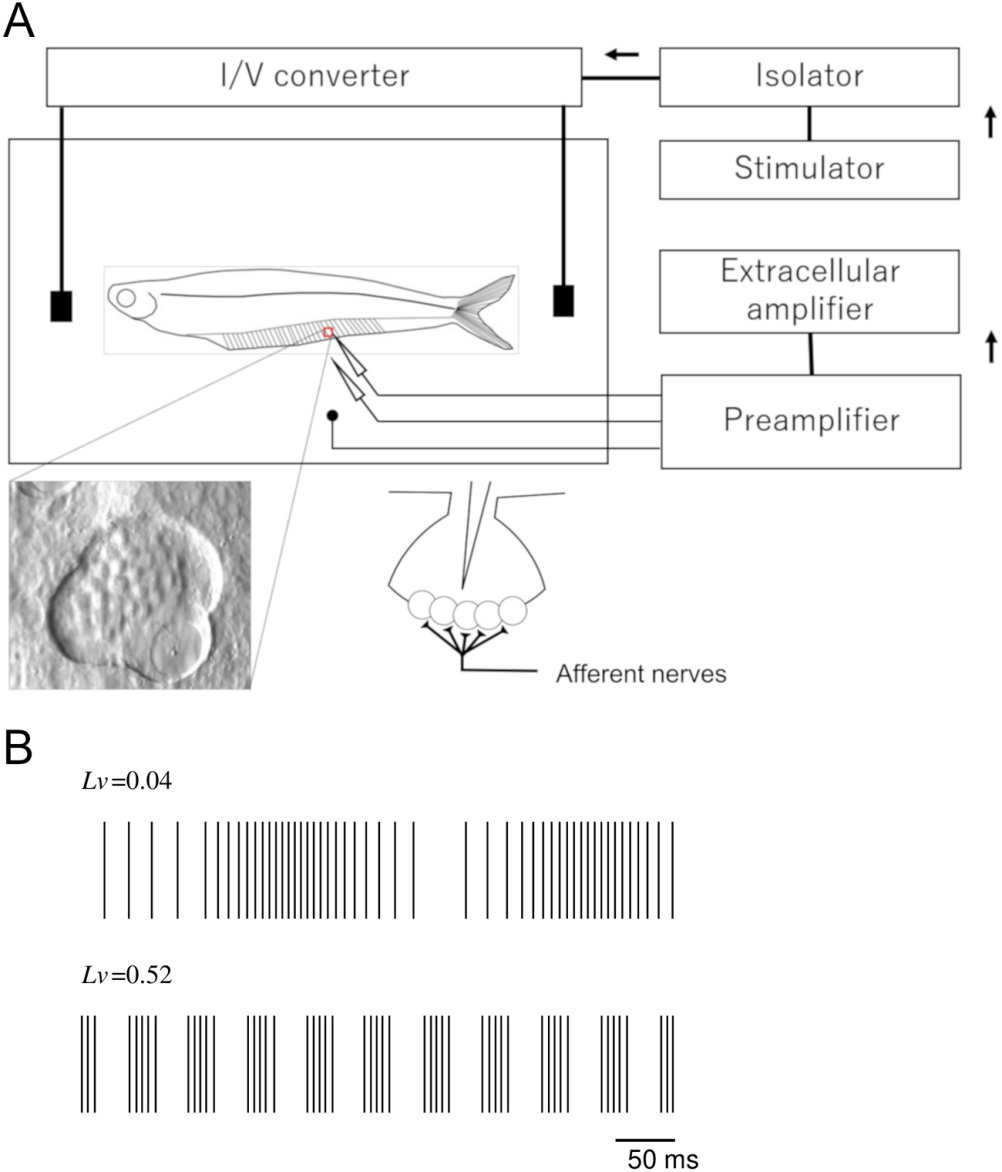
**Experimental installation used in electrophysiological experiments.** (A) Schematic diagram of electrophysiological experiments on electroreceptors. Stimulation electrodes were located near anterior and posterior tips of fish. Sinusoidal electrical stimulation was generated by sine-wave stimulator. Recording electrode was inserted into ampullary canal in anal fin. (B) Spiking pattern modulations and its local variation, *Lv*, which is low when spiking intervals vary gradually. As shown in lower trace, *Lv* is higher when local variation in ISI is large.

Fig. 3A depicts the experimental installation for the electrophysiological recordings. Sinusoidal electrical stimulation was applied to anesthetized glass catfish, and the spikes of the electroreceptors were recorded using an extracellular recording method. The afferent nerves innervated the electroreceptors, as shown in Fig. 3A. Spiking can be recorded extracellularly by inserting an electrode into the ampullary organ. The glass catfish were anesthetized with MS-222 (125 mg/L, 8–12 min of immersion) and placed in an experimental chamber (20.0°C–24.6°C) at room temperature. The perfusion water in the tank was fully oxygenated. A tube was inserted into the oral cavity to allow for gill perfusion. We monitored the migration of the red blood cells in microscopic images to ensure that the anesthetized glass catfish were in proper condition during the experiment. A sinusoidal electric current was passed through a pair of stimulation electrodes placed near the anterior and posterior tips of the fish, whose mean body length was 6.5–10.4 cm (*n*=6). We separately sampled the fish for the electrophysiological experiments from those for the behavioral experiments. The electrode distance was approximately 18 cm. The characteristics of the spontaneous spike rate were consistent with previous works (Bretschneider et al., 1985; Kolomytkin et al., 2007).

**Fig. 4.**
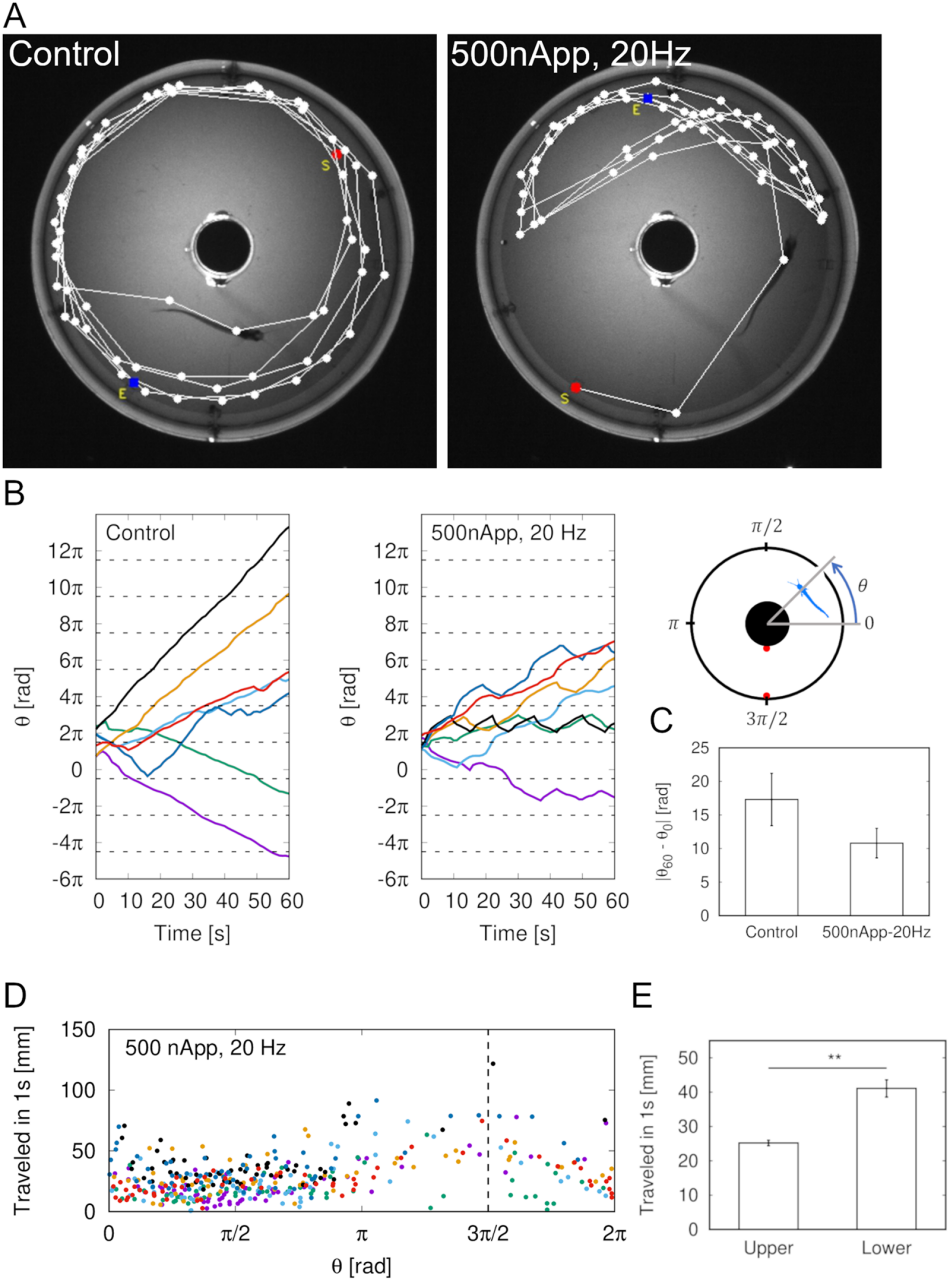
**Fish behavior in response to sinusoidal electrical stimulation.** (A) Locus of fish movements. Dots represent fish locations sampled at 1 Hz. S represents initial location, and E represents location at end of recording. Images show bottom view of aquarium. In no-stimulus condition, fish rotated in aquarium. At 20 Hz and 500-nApp stimulation, fish avoided staying in lower section. (B) Fish position at angle in annular aquarium. Each line represents different fish (*n*=7). Dashed lines indicate location of stimulation electrode. Under control condition, angle θ increased constantly. When stimulated at 20 Hz, fish avoided or hurried past electrode's location. Peak-to-peak amplitude was 500 nApp. (C) Absolute value of angle of stimulus end time minus angle of stimulus start time was statistically tested. θ_0_ is angle at beginning of recording, and θ_60_ is angle at end. Paired *t*-test results show no significant differences by condition, but control group tended to rotate more (*n*=7, *t*(6)=1.276, *P*=0.249). (D) Distance traveled per second around electrode (dashed line) was longer than that around angle π/2. Number of points around electrode was low because there were few opportunities to approach electrode's vicinity. (E) Traveled distance per second in tank's lower half was significantly faster than in upper half [Welch's *t*-test, *n*=338 (upper) and 82 (lower), *t*(96.919)=6.090, *P*<0.001]. **P<0.01.

**Fig. 5.**
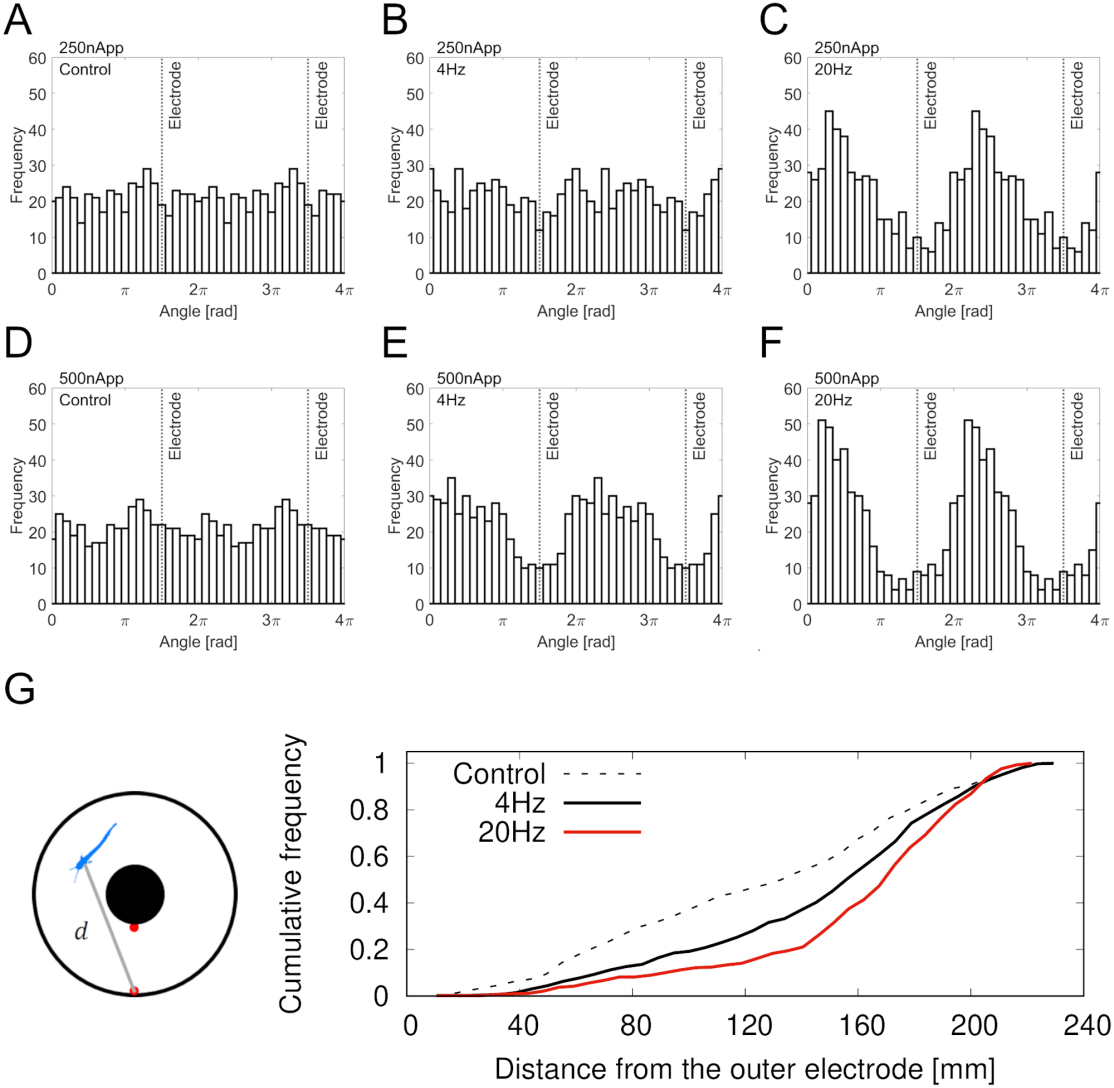
**(A-F) All-point histograms of angles collected from 7 fish for 6 different conditions.** Angle θ is measured in radians. (A,D) In control experiment, fish moved equally in annular aquarium. (B) With 250-nApp stimulation at 4 Hz, fish moved uniformly in annular aquarium. (C) At 250-nApp stimulation, fish stayed in upper section when stimulus frequency was 20 Hz. (E,F) At 500-nApp stimulation, fish mostly stayed in upper section at 4 Hz and 20 Hz. (G) Cumulative distribution of distance from outer electrode. Stimulus amplitude was 500 nApp. Under stimulus conditions, fish were frequently located away from electrodes.

**Fig. 6.**
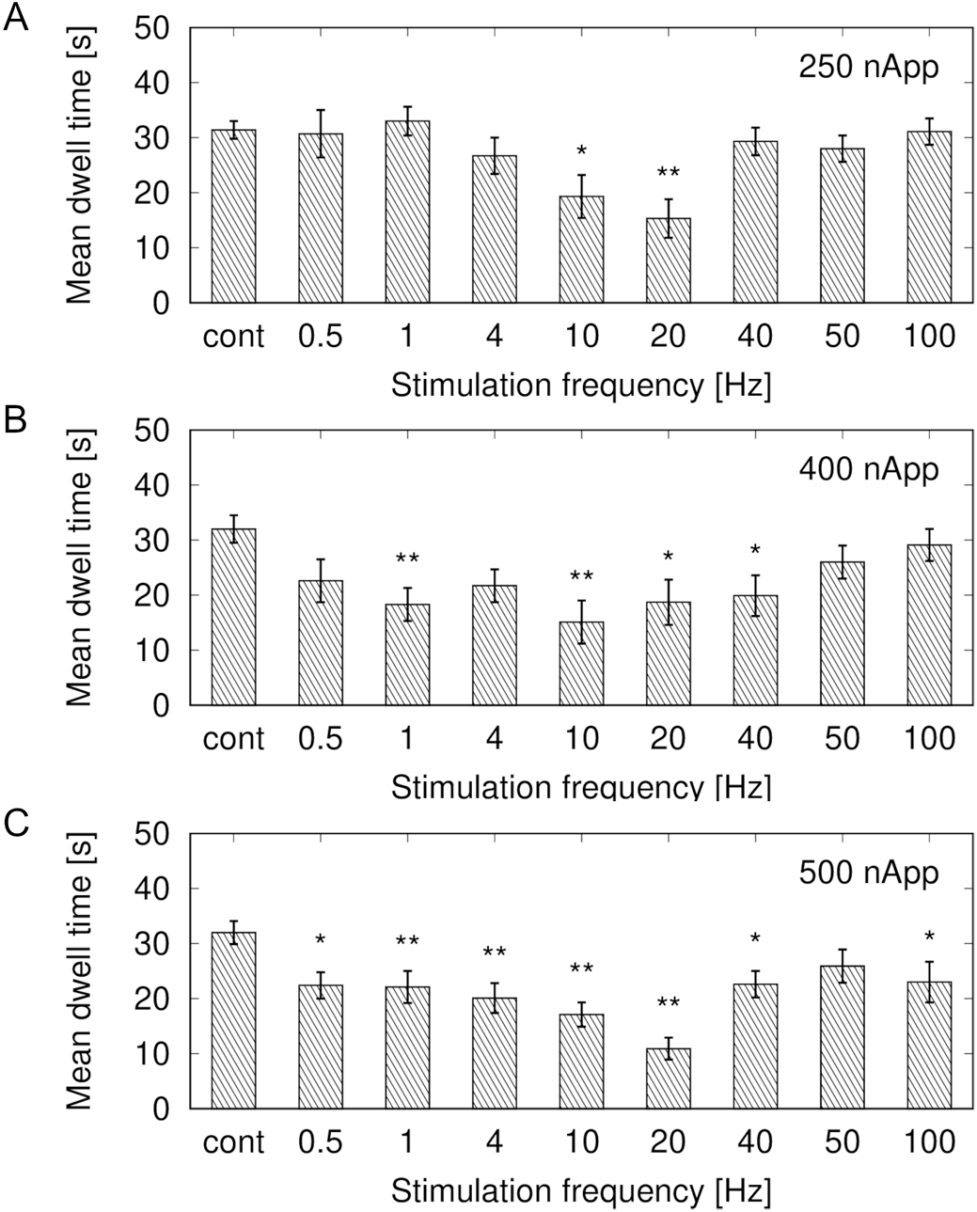
**Mean cumulative dwelling times in lower section during electrical stimulation.** Peak-to-peak current intensities: (A) 250, (B) 400, and (C) 500 nApp. Cont: no electrical stimulation. (A) Mean cumulative dwelling times at 10 Hz and 20 Hz were significantly shorter than that of control group [one-way ANOVA with Dunnett's test, *n*=7, *F*(8, 48)=5.12, *P*<0.001]. No significant difference was found at other frequencies. (B) Low mean cumulative dwelling times were significant in frequency range of 1 Hz and 10–40 Hz [one-way ANOVA with Dunnett's test, *n*=7, *F*(8, 48)=3.746, *P*=0.002]. (C) Mean cumulative dwelling times were significantly lower than that of control group at 0.5–40 Hz and 100 Hz [one-way ANOVA with Dunnett's test, *n*=7, *F*(8, 48)=8.168, *P*<0.001]. ** *P*<0.01, * *P*<0.05.

**Fig. 7.**
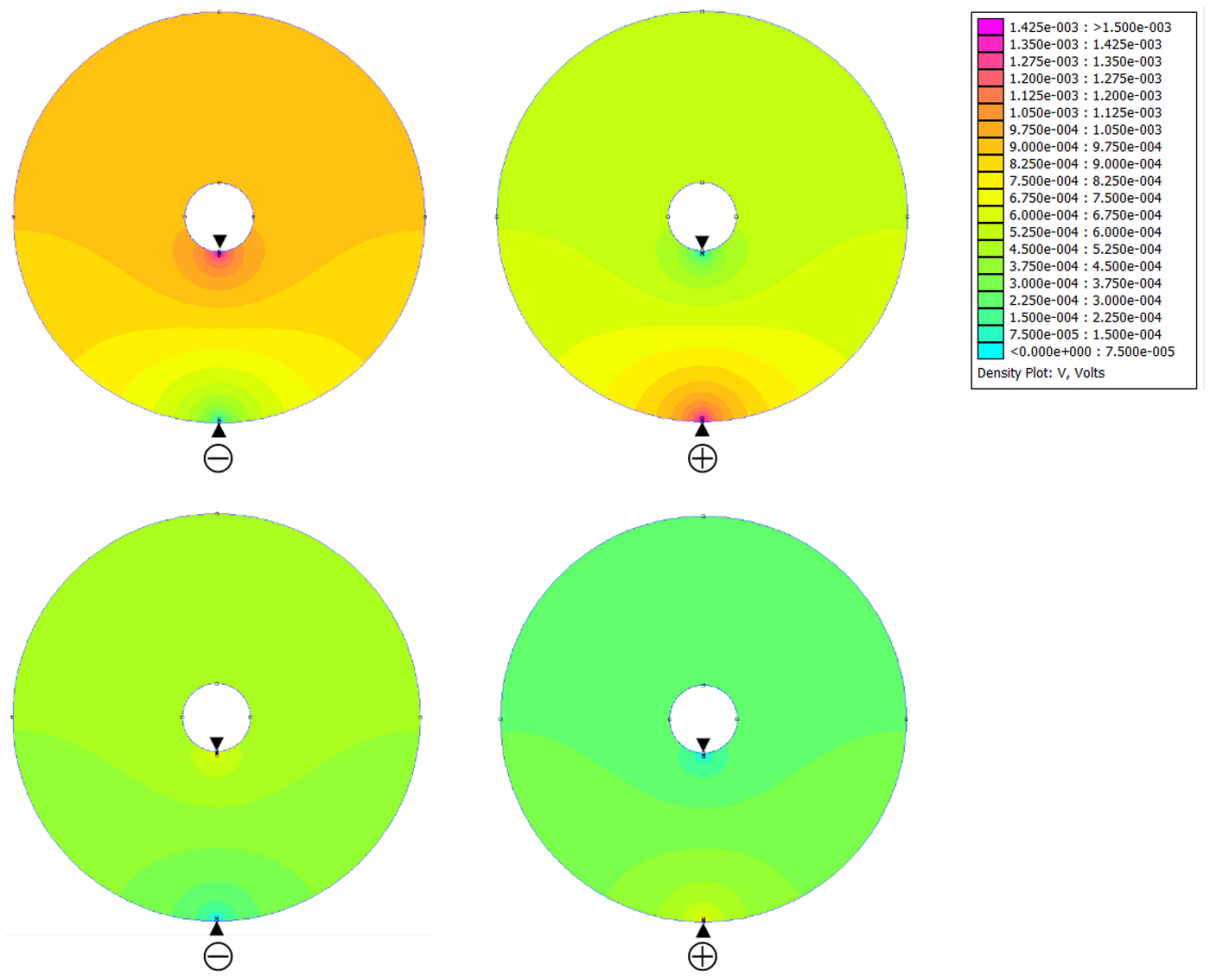
**Electrostatic field generated by stimulation.** Electric potential spread concentrically from electrodes. Electric field decreased with distance from electrodes. Potential gradient near electrodes was steep and became gentle with distance. When cathode potential was high, equipotential lines were densely spaced around electrodes. Arrows indicate position of electrodes.

**Fig. 8.**
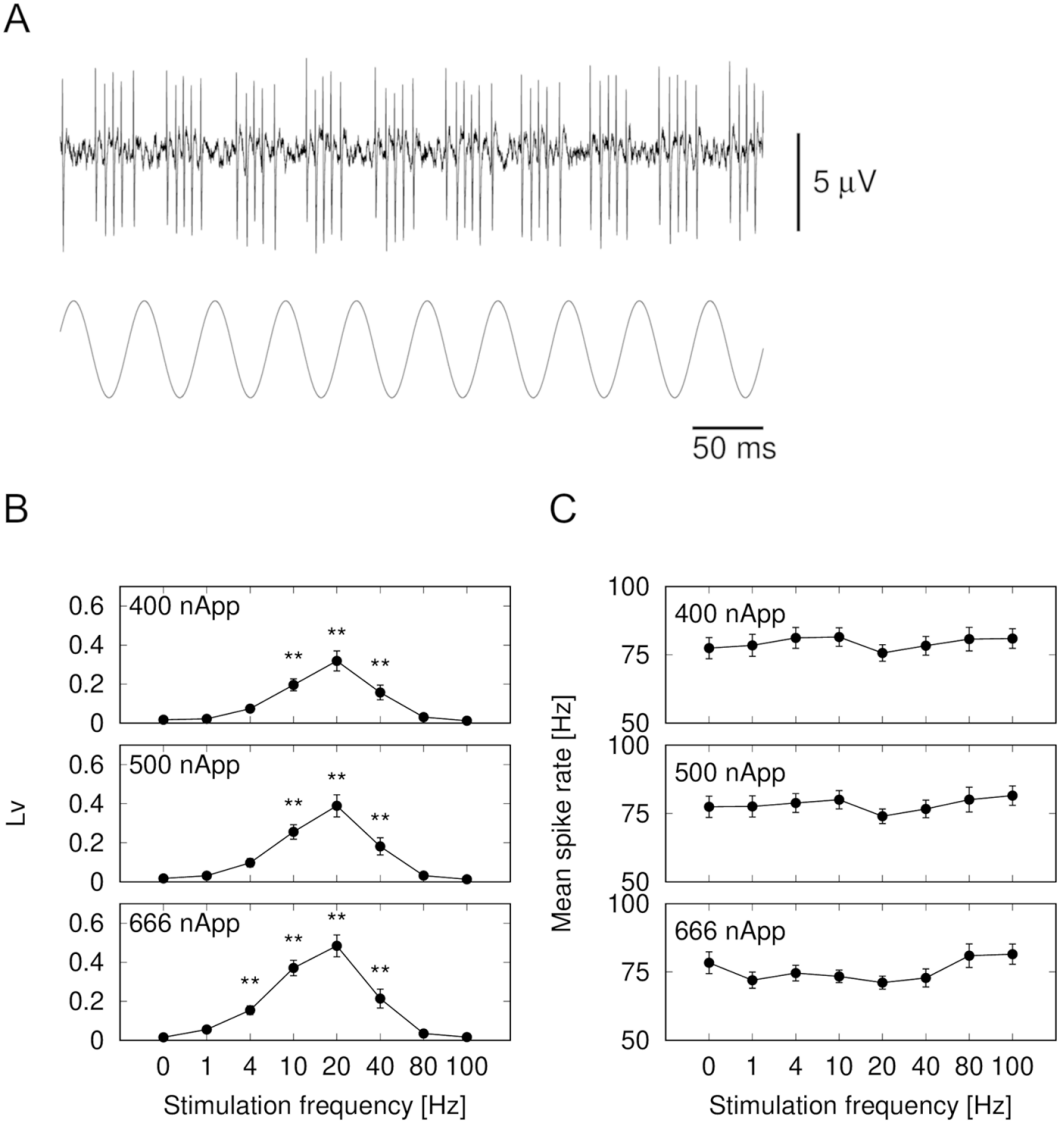
**Frequency modulations of interspike intervals of afferent nerves in response to sinusoidal stimulation.** (A) Bursting pattern to sinusoidal stimulation. Peak-to-peak current amplitude was 500 nApp. (B) Stimulation frequency dependence of *Lv*: For stimulus amplitude=400 nApp, *Lv* was significantly higher in frequency range between 10 and 40 Hz. When stimulus was stronger, *Lv* became larger in low-frequency region of stimulus [one-way ANOVA, *F*(7,140)=22.060, *P<*0.001 at 400 nApp; *F*(7,140)=27.179, *P<*0.001 at 500 nApp; *F*(7,140)=41.148, *P*<0.001 at 666 nApp]. (C) Stimulation frequency dependence of mean spike rate. Mean spike rate depended weakly on stimulation frequency [one-way ANOVA, *F*(7,140)=1.529, *P*=0.162 at 400 nApp; *F*(7,140)=1.482, *P*=0.178 at 500 nApp; *F*(7,140)=3.585, *P*=0.001 at 666 nApp]. Mean±s.e. Data from 21 electroreceptor organs from 6 fish. *P<0.05, **P<0.01.

**Fig. 9.**
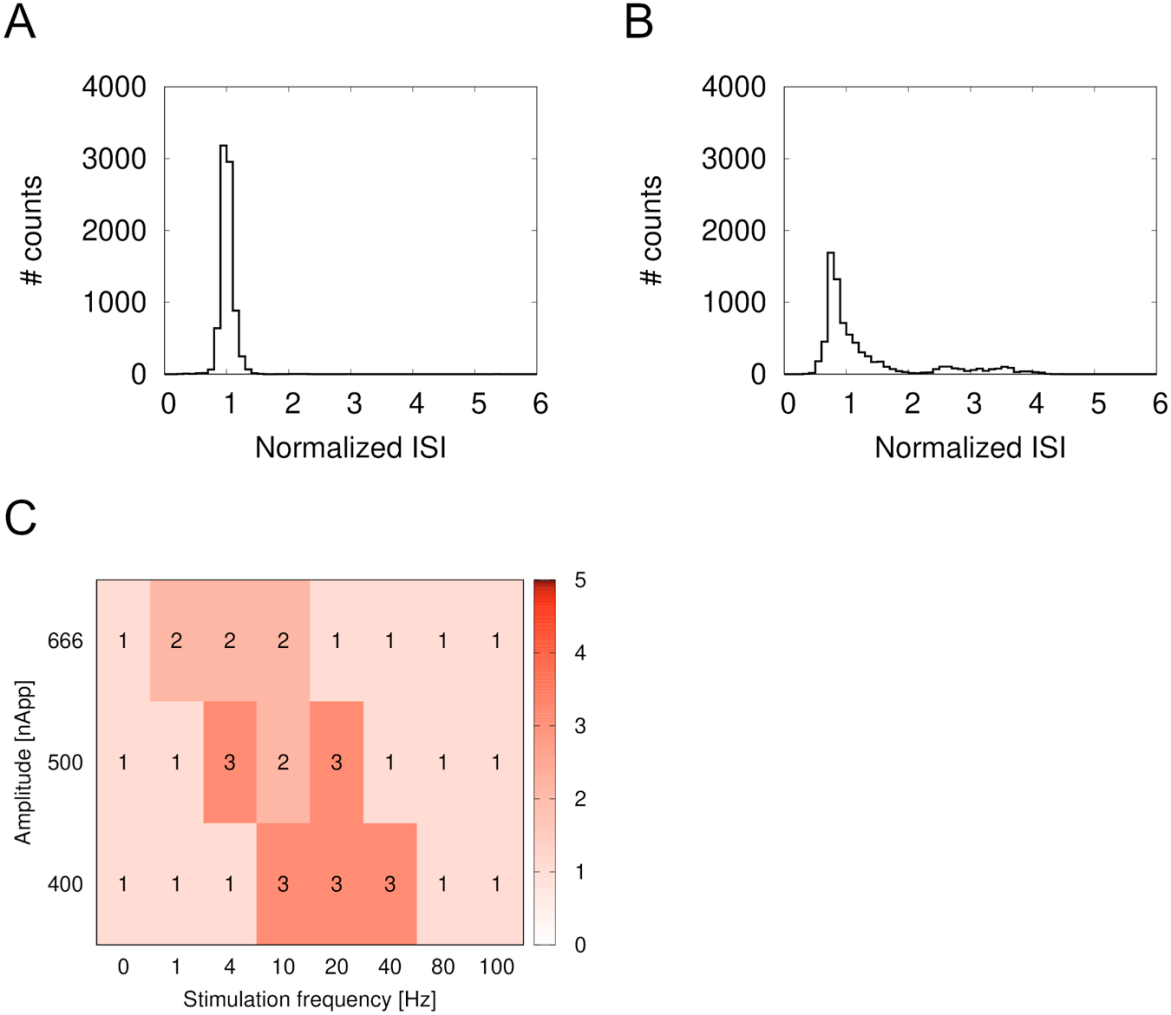
**ISI histograms and number of modes.** ISIs were normalized by mean ISI of spontaneous spiking. (A) Spontaneous spiking. (B) 20 Hz at 500 nApp. Large peak and another broad bump appeared. (C) Heatmap of mode numbers. Number in each box represents mode.

Sinusoidal electrical stimulation was generated by a sine-wave stimulator. We used peak-to-peak current amplitudes of 400, 500, and 666 nApp. The stimulus frequencies were 1, 4, 10, 20, 40, 80, and 100 Hz. Afferent nerve spikes were recorded by inserting a glass microelectrode filled with 50-mM KCl into an ampullary canal of the anal fin. The electric potential was amplified by an extracellular amplifier (DPA1000, Diamedical Systems Co.). To eliminate DC bias fluctuations and high-frequency noise, 0.3-Hz high- and 300-Hz low-pass filters were applied. The filtered signals were sampled at 20 kHz. Spontaneous nerve spikes exhibited regular frequencies with few fluctuations.

#### ISI analysis

The extracellular recording data were processed with Python by using a high-pass filter with a cutoff frequency of 150 Hz. A peak detection algorithm was applied to obtain a series of spike occurrence times from the filtered extracellular data. The peak point was defined as the point that is at least half the height of the 98 percentile point of the data, has a prominence of at least 20%, and has a minimum interval of 5 ms. A spike was defined as a prominence whose height exceeded 45% of the maximum value. ISI is the time interval between two consecutive spikes. ISIs were collected from the filtered data. Next, we assessed the variation of the afferent nerve spikes in the ISIs. Local variation *Lv* (Shinomoto et al., 2003) was introduced to evaluate the ISI variability. *Lv*, which assesses fluctuations in cortical neuron firing and earthquake intervals (Shinomoto et al., 2009; Zhao et al., 2010), is defined as

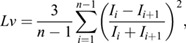
where *I*_*i*_ is the *i*-th ISI, and *n* is the number of ISIs. When ISIs are aligned in short and long alternations, *Lv* increases. *Lv*=0 for a spike train with perfectly regular intervals. *Lv*=1 for a Poisson random series of events with independently exponentially distributed ISIs. Fig. 3B shows the relation between spiking patterns and *Lv*. For a spiking pattern in which the pulse interval varies slowly, *Lv* is low. When periods of rapid spiking are followed by long quiescent periods, *Lv* is high.

The mean spike rate and *Lv* were analyzed using a one-way ANOVA to test for statistical differences between groups. Dunnett's test was then performed for comparisons with controls.
